# Health on the Factory Floor: Occupational Phthalate Exposure Reduces Testosterone

**Published:** 2006-11

**Authors:** Julia R. Barrett

Human studies have shown widespread exposure to phthalates, compounds used in the manufacture of household, consumer, and medical products. The metabolites mono-2-ethylhexyl phthalate (MEHP) and mono-*n*-butyl phthalate (MBP) have shown testicular toxicity in rats, specifically damage to the cells that produce testosterone and sperm. But animal studies involve higher phthalate exposures than humans typically experience, and there is inconclusive evidence that low exposures affect testicular cells in men. Occupational phthalate exposure tends to be greater and more consistent than the highly variable low levels seen in the general population, however, and research at a Chinese manufacturing plant now reveals that such exposure can be significantly related to decreased blood testosterone concentration **[*EHP* 114:1643–1648; Pan et al.]**.

The study participants included 74 men who manufactured polyvinyl chloride (PVC) flooring at a plant in Liaoning Province and 63 men employed at a construction company. All the men completed a questionnaire about lifestyle factors and provided blood and urine samples. Blood samples were analyzed for circulating amounts of free testosterone, luteinizing hormone, follicle-stimulating hormone, and estradiol. Urine analysis provided data on concentrations of MBP and MEHP, which served as biomarkers of exposure.

Due to the materials involved in flooring manufacture, the men at the flooring plant were assumed to have dermal and inhalational exposure to dibutyl phthalate (DBP) and di-2-ethylhexyl phthalate (DEHP), the parent compounds of MBP and MEHP. Indeed, all the participants except one construction worker had detectable levels of urinary MBP and MEHP, demonstrating that phthalate exposure was pervasive. However, PVC plant workers had up to 100-fold higher levels of MBP and MEHP and significantly lower blood testosterone concentrations, compared with construction workers.

Regression analysis revealed a modest but significant decrease in testosterone as total phthalate esters increased. Based on MEHP concentrations, the investigators estimated that 40.5% of the PVC plant workers had DEHP exposure exceeding the European Union’s tolerable daily intake standard of 37.0 μg/kg body weight.

The team concluded that high levels of DEHP and DBP exposure seemed to suppress testosterone production in the PVC plant workers, but it is not clear from this study what effect, if any, that might have on their fertility.

## Figures and Tables

**Figure:**
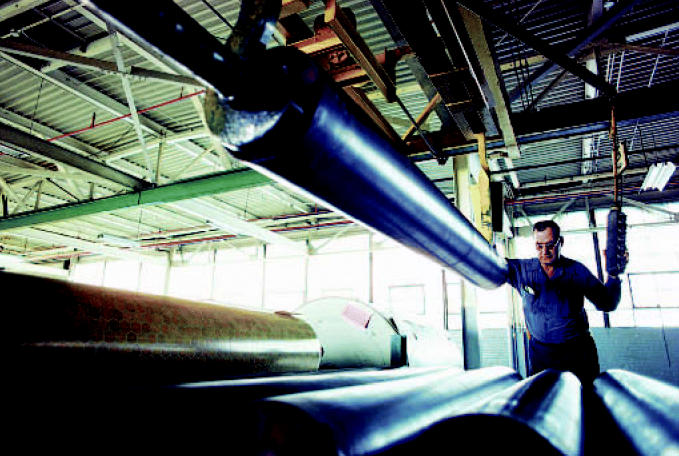
Lowered productivity Men exposed to phthalates in a PVC flooring plant showed decreases in testosterone levels.

